# Adherence to 24‐h movement behaviour guidelines in families with multiple children

**DOI:** 10.1111/cch.13213

**Published:** 2023-12-17

**Authors:** Leigh R. Tooth, Gregore I. Mielke, Katrina M. Moss

**Affiliations:** ^1^ Australian Women and Girls' Health Research Centre, School of Public Health The University of Queensland Herston Queensland Australia

**Keywords:** 24‐h movement behaviour, children, physical activity, screen time, siblings, sleep

## Abstract

**Background:**

In 2019, the World Health Organization (WHO) launched the first global movement guidelines for children that combined sleep, physical activity and screen time. Our previous research showed that adherence to age‐specific guidelines for screen time was challenging for families with children in different age groups. We aimed to determine whether families with children in different age‐based movement guideline categories have poorer adherence to the broader 24‐h movement guidelines than those with all children in the same age category.

**Methods:**

Data were from the 1973–1978 cohort of the Australian Longitudinal Study on Women's Health (seventh survey, 2015) and the women's three youngest children (aged ≤12) (Mothers and their Children's Health sub‐study, 2016/2017). The sample was 1787 women (families) with 4064 children (mean age 7.2 [SD 2.9]). Whether children in the family were in the same or different age‐based category was determined by matching children's ages in a family against age‐based guideline categories for the 24‐h movement behaviours. The association between children in the family being in the same or different age‐based guideline category on adherence to 24‐h movement guidelines, both collectively and individually, was analysed by adjusted logistic regression (binary and multinomial).

**Results:**

Families with children in the same age guideline categories had double the odds of having all children meet 24‐h movement guidelines (adjusted odds ratio [OR] 1.95 [95% confidence interval, CI: 1.32, 2.86]). Families with children in the same age categories on the screen guideline had higher odds of all children meeting (2.25 [1.73, 2.93]) and lower odds of some meeting/some failing the screen guideline (0.18 [0.14, 0.25]), than families with all children in different age categories. Families with children in the same age categories on the physical activity guideline had lower odds of all children meeting (0.57 [0.43, 0.75]) or some meeting/some failing the physical activity guideline (0.08 [0.06, 0.12]). No associations were found for sleep guidelines.

**Conclusions:**

Families with multiple children may need practical advice and strategies on how to adhere to guidelines when children span age categories. This could form part of public health strategies that raise awareness of the guidelines and may improve guideline adherence.

**Key messages**

- In this nested cross‐sectional study of 1787 families (4064 children), families with all children in the same age category were more likely to meet all three 24‐h movement behaviour guidelines (screen time, physical activity and sleep).
- Differing effects were found for meeting the individual screen time and physical activity guidelines by child ages.
- Research into 24‐h movement behaviours, and guideline messaging and parenting resources, should acknowledge the extra challenges faced by families with differently aged children.

## 1 INTRODUCTION

The 24‐h movement guidelines for children and young people, such as those launched in 2019 by the World Health Organization (2019, 2020) and used in Australia and Canada since 2016, recommend physical activity (PA), sleep and sedentary behaviour targets that vary by child age (Australian Department of Health, 2021; Okely et al., 2017; Tremblay et al., 2016). Based on evidence of the short‐ and long‐term harms of not meeting 24‐h movement guidelines in childhood and adolescence, the guidelines aim to promote optimal health and development and reduce children's risk of developing chronic disease in adolescence and beyond (Australian Institute of Health and Welfare, 2018; Biddle et al., 2010; Rollo et al., 2020).

Most studies that assess individual 24‐h movement behaviours (i.e., sedentary behaviour, PA and sleep) use screen time (ST) as the measure of sedentary behaviour, possibly because ST is more easily measurable in minutes or hours per day compared with behaviours like quiet play or reading. Further, guidelines specify time limits for ST as opposed to sedentary behaviour generally. Australian research examining ST, PA and sleep has shown mixed adherence to these individual guidelines (Australian Institute of Health and Welfare, 2020; Olds et al., 2018; Roman‐Viñas et al., 2016; Tooth et al., 2019). Adherence to ST guidelines is poorer in older children (26% in ages 10–14) than younger children (39% in ages 5–9) (Australian Institute of Health and Welfare, 2020). Similarly, only 23% of Australian children aged 5–14 years perform the recommended PA. When compared by age, 32% of those aged 5–9 adhere to PA guidelines compared with 15% of those aged 10–14. Adherence to sleep guidelines is higher (Grady et al., 2018; Scully et al., 2022), with 90% of children aged 11 and 70% of those aged 14–15 meeting guidelines on school nights, although it drops to 50% by ages 16–17 (Evans‐Whipp & Gasser, 2019). Poor adherence to guidelines is not unique to Australia, being also reported in North America, Canada and across Asian, African and European countries (Friel et al., 2020; Gomes et al., 2017; Hui et al., 2021; Poitras et al., 2016; Roberts et al., 2017).

The premise behind the 24‐h movement guidelines is that adherence of these individual movement behaviours in combination is associated with better health (Okely et al., 2022; Roman‐Viñas et al., 2016). In two Australian studies of children aged 9–11, adherence to 24‐h movement guidelines was 4%–16% (Olds et al., 2018; Roman‐Viñas et al., 2016), with 3%–15% meeting none and 22%–52% and 29%–60% meeting one or two guidelines, respectively (Olds et al., 2018). A study of 12 countries (Australia, Brazil, Canada, China, Colombia, Finland, India, Kenya, Portugal, South Africa, the United Kingdom and the United States) found similar results (Sampasa‐Kanyinga et al., 2017). Differences in adherence to the 24‐h movement guidelines have been found by child age (poorer with increased age), sex (poorer in girls) and socio‐economic factors (poorer in countries with lower Human Development Indices) (Tapia‐Serrano et al., 2022).

To understand adherence to 24‐h movement guidelines, researchers must consider the family context. Many families have multiple children, yet research on guideline adherence typically only includes one child from each family and does not account for when siblings are in different age‐based categories. In our study of 4543 children aged 0–12 from 1993 families (up to three children per family), 54% of families with children in the same age‐based ST category met ST guidelines compared with 23% of families with children in different age‐based ST categories (Tooth et al., 2022). This suggests that the ages of the children within the family may influence ST guideline adherence. Currently, it is unknown whether adherence to the broader 24‐h movement guidelines also differs by whether children span age‐based guidelines. Research designs including more than one child from a family may answer this question.

The aim of this study was to investigate whether families with children in different age‐based guideline categories have poorer adherence to all three 24‐h movement guidelines than those with all children in the same age category. The secondary aim of the study was to investigate the association between having children in different age‐based guideline categories and adherence to the three individual components of the 24‐h movement guidelines (ST, PA and sleep), compared with those with all children in the same age category.

## 2 METHODS

### 2.1 Study design and participants

**FIGURE 1:**
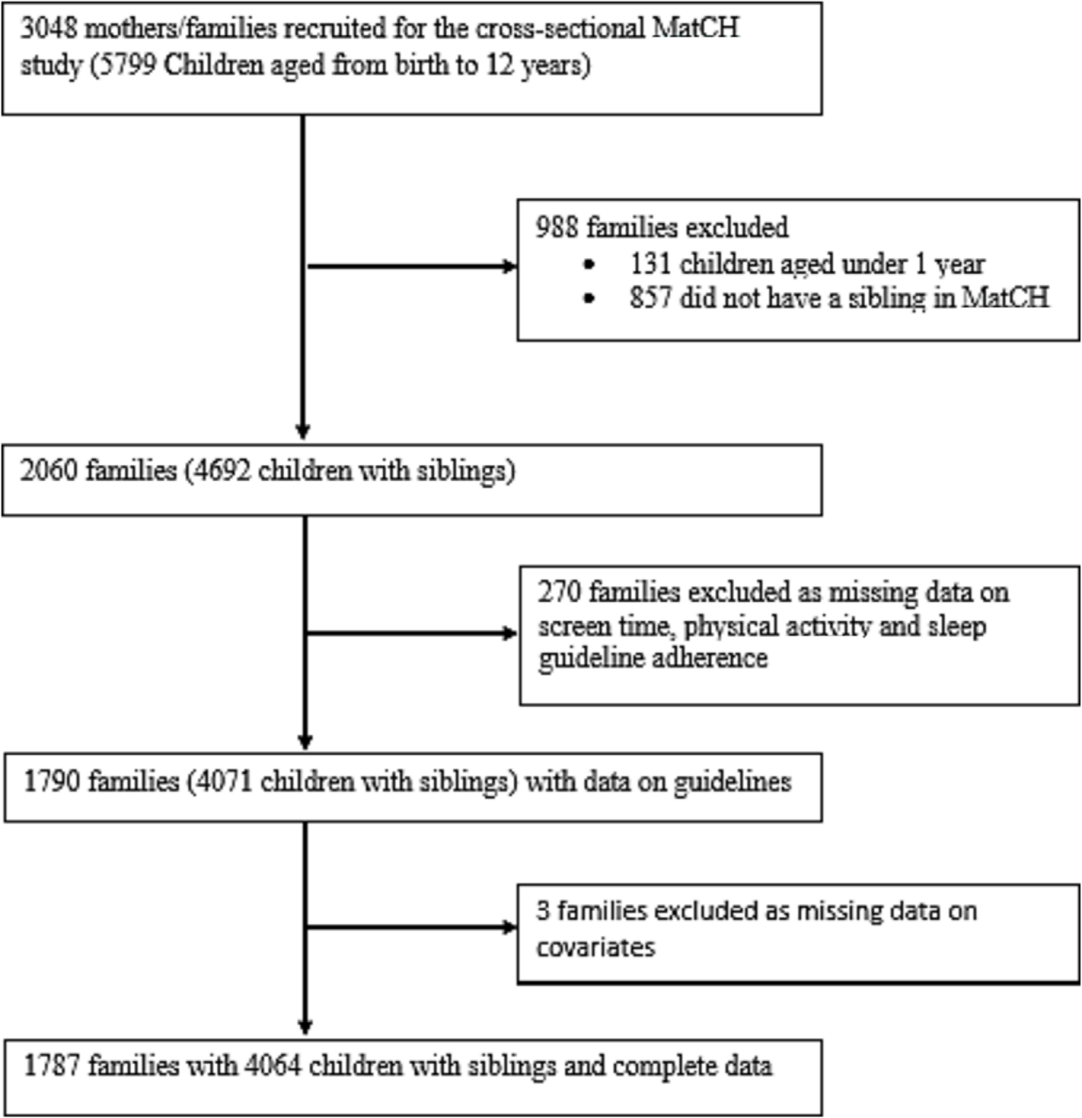
Study flow diagram. MatCH, Mothers and their Children's Health.

Data were from the Mothers and their Children's Health (MatCH) study (Mishra et al., 2018), a nested cross‐sectional sub‐study of the Australian Longitudinal Study on Women's Health (ALSWH), which is broadly representative of Australian women born between 1989–1995, 1973–1978, 1946–1951 and 1921–1926 (Dobson et al., 2015). In 2016, ALSWH participants in the 1973–1978 cohort were invited to take part in a sub‐study of their three youngest children aged ≤12 (i.e., born from 2004 onwards). Eligible participants were those who had not died or withdrawn, had consented to be contacted about sub‐studies and had not reported infertility (Mishra et al., 2018). Approximately 48% of eligible mothers participated. Participants were more likely to be university educated, employed and non‐smokers than nonparticipants (Mishra et al., 2018). MatCH study data were collected from August 2016 to May 2017. Data on covariates from the mothers were collected at the ALSWH survey prior to MatCH (in 2015). Both studies were approved by the Human Research Ethics Committees at the University of Queensland and University of Newcastle in Australia. Mothers provided informed written consent for all surveys in both the ALSWH and MatCH studies. All data collected in both surveys were provided by the mothers. For this study, we excluded children without siblings in MatCH, those aged under 1 year (as we could not measure PA guidelines) and those without complete data on all three guidelines (total exclusions *N* = 1261; Figure 1 and Table 1).

**TABLE 1:** Included and excluded families from original MatCH study.

Mother (family) level characteristics, *N* (%)	Included (*N* = 1787)	Eligible but excluded due to missing data^a^ (*N* = 273)	Ineligible other MatCH families^a^ (*N* = 988)
Age of mothers, mean (SD, range)	40.3 (1.46, 37–43)	40.4 (1.49, 38–44)	40.4 (1.52, 37–43)
Age of children, mean (SD, range)	7.20 (2.91, 1–12)^b^	7.00 (3.05, 1–12)^c^	6.32 (4.16, 0–12)^d^
One child	0	0	896 (90.7)
Two children	1297 (72.6)	191 (70.0)	65 (6.6)
Three children	490 (27.4)	82 (27.3)	27 (2.7)
Mother's education			
≤ Year 12	205 (11.5)	42 (15.4)	172 (17.4)
Trade/certificate/diploma	372 (20.8)	67 (24.5)	278 (28.1)
Degree/higher degree	1210 (67.7)	164 (60.1)	538 (54.5)
Mother partnership status			
Partnered	1688 (94.5)	254 (93.0)	816 (82.6)
Unpartnered	99 (5.5)	19 (7.0)	172 (17.4)
Area of residence			
Metropolitan	1081 (60.5)	168 (61.5)	577 (58.4)
Inner regional	455 (25.5)	64 (23.4)	258 (26.1)
Outer regional/remote	251 (14.1)	41 (15.0)	153 (15.5)
Mother exercise			
Very low	232 (13.0)	45 (16.9)	142 (14.4)
Low	581 (32.5)	84 (31.6)	311 (31.5)
Moderate	408 (22.8)	56 (21.1)	234 (23.7)
High	566 (31.7)	81 (30.4)	299 (30.3)
Missing	0	7	2
Mother BMI			
Underweight/healthy weight	958 (53.6)	142 (53.2)	465 (47.3)
Overweight	489 (27.4)	78 (29.2)	266 (27.1)
Obese	340 (19.0)	47 (17.6)	251 (25.6)
Missing	0	6	6
Mother depressive symptoms			
No	1487 (83.2)	208 (76.2)	727 (73.6)
Yes	300 (16.8)	65 (23.8)	261 (26.4)
Biological sex of children			
Girls only	310 (17.4)	51 (19.3)	355 (36.0)
Boys only	395 (22.1)	57 (21.5)	324 (32.9)
Both girls and boys	1082 (60.6)	157 (59.3)	307 (31.1)
Missing	0	8	2
Family has ≥1 child with electronic (screen) equipment in bedroom			
No	1116 (62.5)	79 (68.7)	698 (63.5)
Yes	671 (37.5)	36 (31.3)	402 (36.5)
Missing	0	158	7

Abbreviations: BMI, body mass index; MatCH, Mothers and their Children's Health.

^a^ See Figure 1 for details.

^b^ *N* = 4064 children.

^c^ *N* = 628 children.

^d^ *N* = 1107 children.

### 2.2 Measures

#### 2.2.1 Exposures

For the primary aim, the exposure was whether all children in the family were in the same age‐based category on all three guidelines (yes or no). This was determined by matching children's ages in a family against age‐based guideline categories for the 24‐h movement behaviours. For the second aim, whether all the children in the family were in the same age‐based category was calculated individually for ST, PA and sleep guidelines (each scored yes or no).

#### 2.2.2 Outcomes

Adherence to 24‐h movement guidelines was determined by matching mother self‐report of children's ST, PA and sleep behaviours to the age‐based guidelines for those behaviours. For ST, mothers reported average time per day over the last month children spent watching or using any screen‐based equipment (i.e., televisions, computers, tablets, mobile phones or electronic games) on weekdays and weekends (in hours or minutes). Children met guidelines if they had no time per day if aged <2, ≤1 h/day if aged 2–4 and ≤2 h if aged 5 and older. For PA, mothers reported on how many days in a typical week their child engaged in at least 3 h/day in active play (if aged 1–4) or ≥60 min of moderate to vigorous PA/day (if aged ≥5 years) (Okely et al., 2017). Children were adherent if they spent ≥3 h in active play every day (for children aged 1–4) or ≥60 min of moderate to vigorous PA every day (if aged ≥5 years). Mothers also reported the number of hours or minutes of sleep the child had during the day and night. Children met sleep guidelines if they had 11–14 h/day if aged ≤2, 10–13 h/day if aged 3–5 and 9–11 h/day if aged >5 years (Okely et al., 2017). Table 2 summarises how adherence to guidelines was determined in the study and how they compare with Australian guidelines. For the primary aim, the outcome was whether all children in the family met the age‐based guidelines for all three guidelines (scored yes or no). For the secondary aim, outcomes were whether all children in the family met the age‐based guidelines for each 24‐h movement behaviour individually: (a) ST, (b) PA and (c) sleep (scored as all children in the family meet the guidelines, some children in the family meet/some fail the guidelines and all children in the family fail the guidelines).

**TABLE 2:** Methods used to determine adherence to Australian 24‐h movement guidelines in the current study.

Australian government 24‐h movement guidelines (Australian Department of Health, 2021)		Questions asked in MatCH survey	How adherence to guidelines was determined in the MatCH study
Recreational screen time			
Ages 0–1	No time.	Over the past month, about how much screen time has your child had per day? (*Screen time includes time spent watching/using any screen‐based equipment such as television, computers, tablets, mobile phones and electronic games*.) Recorded separately to the nearest 15 min for weekdays and weekend days. Recorded separately for school work/homework and for all other reasons.	Children met guidelines if they had no time per day if aged <2 ≤1 h/day if aged 2–4 ≤2 h/day if aged 5 and older.
Ages 2–4	1 h/day.		
Ages 5–12	2 h/day.		
Physical activity			
Ages 0–1	Babies should be physically active several times a day in various ways.	No equivalent questions asked.	
Ages 1–2	At least 3 h of various physical activities each day, including energetic play.	Over the past week, on how many days did your child spend a total of at least 3 h/day in active play? AND Over a typical or usual week, on how many days did your child spend a total of at least 3 h/day in active play? (*Active play is any physical activity that your child does. Some examples of active play are walking, running, climbing, rising a tricycle or scooter, swimming, dancing and ball games*.)	Children met guidelines if they spent ≥3 h in active play every day if aged 1–4 years ≥60 min of moderate to vigorous PA every day if aged ≥5 years.
Ages 3–5	Active for at least 3 h each day. This should include 1 h of energetic play.		
Ages 5–12	At least 1 h/day of moderate to vigorous physical activity. At least 3 days/week, incorporate vigorous activities and activities that strengthen muscle and bone in the 1 h.	Over the past week, on how many days did your child spend a total of at least 1 h/day in moderate to vigorous physical activity? AND Over a typical or usual week, on how many days did your child spend a total of at least 1 h/day in moderate to vigorous physical activity? (*Moderate to vigorous physical activity is any form of physical activity that includes bursts of high energy, raises your child's heart rate and makes them ‘huff and puff’*.)	
Sleep			
Ages 0–1	16–17 h/day.	How much time does your child spend in sleep? (*Recorded in minutes and hours to the nearest 15 min*.)	Children met sleep guidelines if they had 11–14 h/day if aged ≤2 years 10–13 h/day if aged 3–5 years 9–11 h/day if aged >5 years.
Ages 1–2	11–14 h/day.		
Ages 3–5	10–13 h/day.	What is your child's usual amount of sleep each day (combining night time sleep and naps)? (*Recorded in minutes and hours to the nearest 15 min*.)	
Ages 5–12	9–11 h/day.		

Abbreviations: MatCH, Mothers and their Children's Health; PA, physical activity.

#### 2.2.3 Covariates

Covariates were chosen from research with the current data that examined adherence to ST guidelines (Tooth et al., 2019, 2022), as well as a literature review of factors associated with adherence with 24‐h movement guidelines. Maternal covariates were area of residence (major city, inner regional, outer regional or rural/remote) (Glover & Tennant, 2003), partnership status (partner or no partner), highest educational qualification (up to grade 12, trade/certificate/diploma or degree/higher degree), body mass index (underweight/healthy weight, overweight or obese) (World Health Organization, 2000), level of PA (categorised as nil, low, moderate or high) (Brown et al., 2005) and depressive symptoms measured using the 10‐item Centre for Epidemiology Studies Depression Scale‐10, with a score of ≥10 indicating depressive symptoms (Andresen et al., 1994). Child covariates were sex and electronic equipment in the child's bedroom (yes or no) (Table S1).

### 2.3 Statistical analysis

Descriptive statistics were calculated to check cell sizes. Chi‐square tests were used to compare the characteristics of included, eligible‐but‐excluded (due to missing; Figure 1) and ineligible families. Binary logistic regression was used to examine the association between age categories within families and meeting the 24‐h movement guidelines. For the individual ST and PA guidelines, multinomial logistic regression was used to examine associations between age categories within families and adherence. For sleep, there was not enough variability in the data for a three‐level outcome variable, so the outcome was dichotomised into meet guidelines/did not meet guidelines and bivariate logistic regression was used. For both analyses, a crude model without covariates was run first (Model 1). Covariates were added in steps: Model 2 added maternal education, area of residence and partnership status; Model 3 added sexes of children; Model 4 added maternal body mass index, PA and maternal depression; and Model 5 added electronic equipment in child's bedroom. To confirm that family‐level effects were not an artefact of the children's ages, sibling effects were tested by stratifying the children by age (0–4 or 5–12 years) and examining adherence by age of siblings. Analyses were conducted using SAS software (Version 9.4) (SAS Institute Inc., 2016).

## 3 RESULTS

**FIGURE 2:**
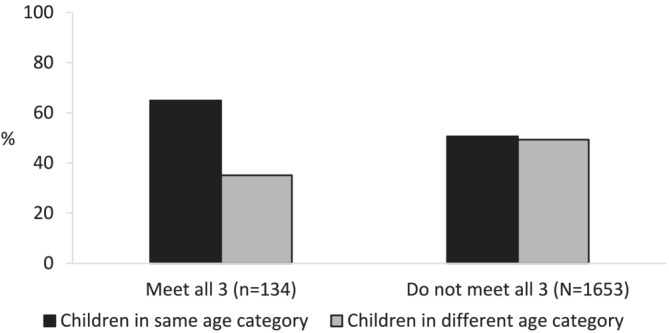
Adherence to all three 24‐h movement guidelines by whether all children are in the same age‐based guideline category for all three guidelines.

Of 2060 eligible families, 1787 families (4064 children) were included (Figure 1). Most families had two children (73%), had both boys and girls (61%), lived in metropolitan areas (61%) and had a university‐educated mother (68%) (Table 1). Overall, 134 (8%) families had children meeting all 24‐h movement guidelines, 755 (42%) met two, 775 (43%) met one and 123 (7%) met none. Of families meeting all three 24‐h movement guidelines, 65% had children in the same age‐based category for each guideline, compared with 35% with children in different age‐based categories (Figure 2). The adjusted regression models confirmed this association; families with children in the same age‐based guideline categories had double the odds (odds ratio [OR] 1.95 [95% confidence interval, CI: 1.32, 2.86]) of having all children meet the 24‐h movement guidelines (Table 3).

**TABLE 3:** The association between whether families have children in the same age‐based guideline category and adherence to all three 24‐h behaviour guidelines in 1787 families.

Exposure	Model 1, OR (95% CI)	Model 2, OR (95% CI)	Model 3, OR (95% CI)	Model 4, OR (95% CI)	Model 5, OR (95% CI)
Children in same age‐based guideline categories for all 3 guidelines (*n* = 863 families)	**1.81 (1.25, 2.61)**	**1.82 (1.26, 2.64)**	**1.81 (1.24, 2.62)**	**1.67 (1.14, 2.43)**	**1.95 (1.32, 2.86)**
Children in different age‐based guideline categories for all 3 guidelines (*n* = 924 families)	Ref	Ref	Ref	Ref	Ref

*Notes*: Model 1: Exposure = same or different age categories; Model 2: Model 1 + maternal education, partnership status and area of residence; Model 3: Model 2 + sexes of children; Model 4: Model 3 + maternal exercise, body mass index and depression; and Model 5: Model 4 + child bedroom electronic equipment. Boldface indicates statistical significance (*p* < 0.05).

Abbreviations: CI, confidence interval; OR, odds ratio; Ref, reference.

**FIGURE 3:**
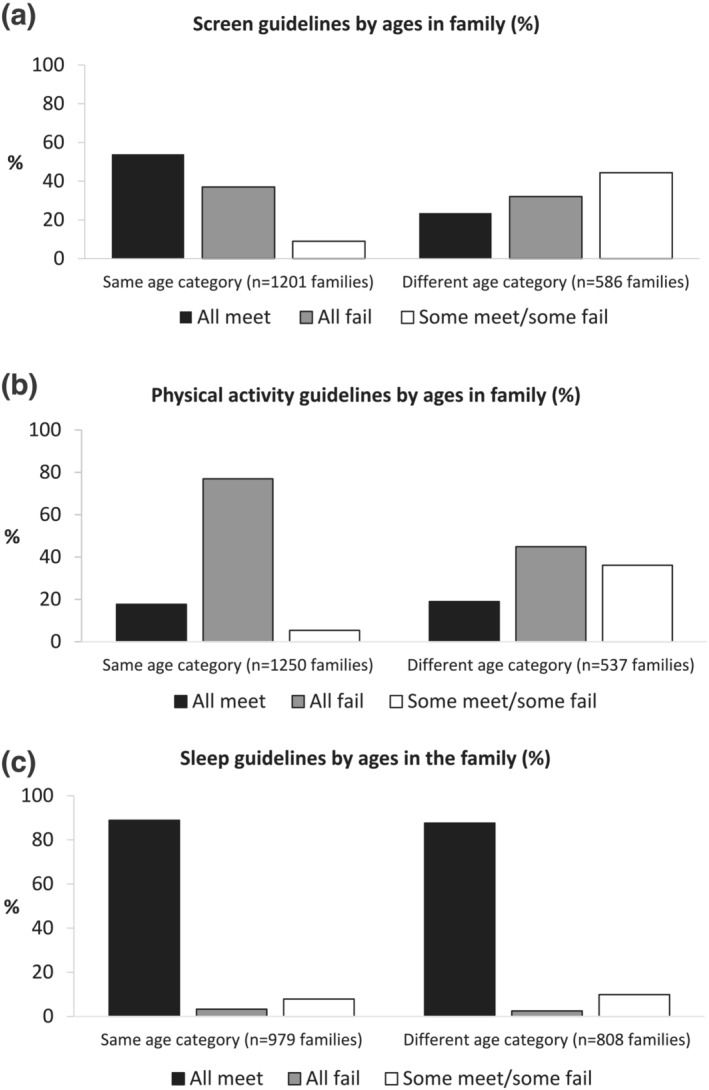
Adherence to (a) screen, (b) physical activity and (c) sleep guidelines by whether all children are in the same age‐based category for that guideline.

Individually, ST, PA and sleep guidelines were met by all children in 44%, 18% and 88% of families, respectively. Adherence to each 24‐h movement varied widely by age categories of the children (Figure 3). Descriptively, more families with all children in the same ST age category had all children meeting the ST guideline (54%) and very few who were discrepant (9%) (Figure 3a), compared with those with children in different ST categories (23.5% and 44%, respectively). The adjusted regression model showed that families with children in the same ST age categories had twice the odds of all children meeting (2.25 [1.73, 2.93]) and 82% lower odds of some meeting/some failing the ST guideline (0.18 [0.14, 0.25]; Table 3), versus families with all children in different age categories.

More families with children in the same PA age category did not meet the PA guideline than those with children in different age categories (77% vs. 45%) while discrepancy was higher in families with children in different age categories versus same age categories (36% vs. 5%) (Figure 3b). The adjusted regression model showed that families with children in the same PA age categories had 43% lower odds of all children meeting (0.57 [0.43, 0.75]) and 92% lower odds of some meeting/some failing the PA guideline (0.08 [0.06, 0.12]; Table 4), versus families with all children in different age categories.

**TABLE 4:** The association between whether families have children in the same age‐based guideline category and adherence to the screen time and physical activity components of the 24‐h movement guidelines in 1787 families.

	Model 1, OR (95% CI)		Model 2, OR (95% CI)		Model 3, OR (95% CI)		Model 4, OR (95% CI)		Model 5, OR (95% CI)	
Exposures	All meet guideline	Some meet/some fail guideline	All meet guideline	Some meet/some fail guideline	All meet guideline	Some meet/some fail guideline	All meet guideline	Some meet/some fail guideline	All meet guideline	Some meet/some fail guideline
Screen time guidelines										
Children in same age‐based screen guideline category (*n* = 1201 families)	**1.98 (1.54, 2.55)**	**0.18 (0.13, 0.23)**	**2.12 (1.64, 2.73)**	**0.18 (0.14, 0.24)**	**2.12 (1.65, 2.74)**	**0.18 (0.14, 0.24)**	**2.02 (1.56, 2.62)**	**0.18 (0.14, 0.24)**	**2.25 (1.73, 2.93)**	**0.18 (0.14, 0.25)**
Children in different age‐based screen guideline category (*n* = 586 families)	Ref	Ref	Ref	Ref	Ref	Ref	Ref	Ref	Ref	Ref
Physical activity guidelines										
Children in same age‐based physical activity guideline category (*n* = 1250 families)	**0.54 (0.41, 0.71)**	**0.09 (0.06, 0.12)**	**0.55 (0.42, 0.72)**	**0.09 (0.06, 0.12)**	**0.54 (0.41, 0.71)**	**0.09 (0.06, 0.12)**	**0.52 (0.39, 0.69)**	**0.08 (0.06, 0.11)**	**0.57 (0.43, 0.75)**	**0.08 (0.06, 0.12)**
Children in different age‐based physical activity guideline category (*n* = 537 families)	Ref	Ref	Ref	Ref	Ref	Ref	Ref	Ref	Ref	Ref

*Note*: Model 1: Unadjusted; Model 2: Model 1 + maternal education, partnership status and area of residence; Model 3: Model 2 + sexes of children; Model 4: Model 3 + maternal exercise, body mass index and depression; and Model 5: Model 4 + child bedroom electronic equipment. Boldface indicates statistical significance (*p* < 0.05).

Abbreviations: CI, confidence interval; OR, odds ratio; Ref, reference.

Almost all the children met the sleep guideline, regardless of whether they were in the same or different age categories (Figure 3c). The regression analyses confirmed the lack of association between age category and adherence (unadjusted 1.13 [0.84, 1.51]; adjusted 1.37 [1.01, 1.85]; Table 5).

**TABLE 5:** Having children in the same age‐based sleep guideline category and adherence to the sleep component of the 24‐h movement guidelines.

Exposure	Model 1, OR (95% CI)	Model 2, OR (95% CI)	Model 3, OR (95% CI)	Model 4, OR (95% CI)	Model 5, OR (95% CI)
Children in same age‐based sleep guideline category (*n* = 979 families)	1.13 (0.84, 1.51)	1.17 (0.88, 1.56)	1.18 (0.89, 1.58)	1.17 (0.87, 1.57)	**1.37 (1.01, 1.85)**
Children in different age‐based sleep guideline category (*n* = 808 families)	Ref	Ref	Ref	Ref	Ref

*Note*: Model 1: Exposure = same or different age categories; Model 2: Model 1 + maternal education, partnership status and area of residence; Model 3: Model 2 + sexes of children; Model 4: Model 3 + maternal exercise, body mass index and depression; and Model 5: Model 4 + child bedroom electronic equipment. Boldface indicates statistical significance (*p* < 0.05).

Abbreviations: CI, confidence interval; OR, odds ratio; Ref, reference.

In the sensitivity analysis, sibling effects were found for ST for children aged 0–4 (adherence better if no older sibling). For PA, adherence was better if children aged 0–4 had no older or same‐aged sibling and better for children aged 5–12 with a younger sibling.

## 4 DISCUSSION

In this family‐based study, we investigated whether families with children in different age‐based guideline categories have poorer adherence to all three 24‐h movement guidelines than those with all children in the same age category. For the guidelines overall, if children were in the same age‐based guideline category, adherence was higher. However, no consistent association between ages of children in a family and adherence to all three individual 24‐h movement behaviour guidelines was found. For ST, if children were in the same age‐based guideline category, adherence was higher and adherence discrepancy between children in the family was less. For PA, overall adherence was poor regardless of the ages of the children but, similarly to ST, adherence discrepancy between children in the family was less when children were in the same age category. For sleep, overall adherence was very high, regardless of the children's ages.

Only 8% of families in our study had all children meeting the three 24‐h movement guidelines. Comparison of these results with other studies is difficult as our study included children as young as 1 year, compared with others that focus on children in middle childhood and early adolescence. A systematic review of over 387 000 children and adolescents aged between 3 and 18 years found overall that 7% met ST, PA and sleep guidelines, with adherence to all three guidelines ranging from 11.2% in preschool children to 3% in adolescents (Tapia‐Serrano et al., 2022). Our finding that 43% and 42% of Australian families had all children meeting one or two guidelines, respectively, is also concerning given findings that the more guidelines being met (irrespective of type), the better the physical, mental and social health indicators in children (Janssen et al., 2017).

Consistent with other research (Australian Institute of Health and Welfare, 2020; Evans‐Whipp & Gasser, 2019; Friel et al., 2020; Gomes et al., 2017; Grady et al., 2018; Hui et al., 2021; Olds et al., 2018; Roberts et al., 2017; Roman‐Viñas et al., 2016; Scully et al., 2022; Tooth et al., 2019), adherence to individual guidelines varied by movement behaviour, with 44%, 18% and 88% of families having all children meeting ST, PA and sleep guidelines, respectively. Adherence to sleep guidelines tends to be the highest of the three behaviours, particularly in younger children (Grady et al., 2018; Scully et al., 2022), possibly reflecting the biological necessity of sleeping and that it tends to occur within the home. Meeting ST and PA guidelines has been found to range from 5% to 87% (Cooper et al., 2015; Gomes et al., 2017; Olds et al., 2018; Roman‐Viñas et al., 2016; Telford et al., 2013). Our finding that adherence to PA or sleep guidelines was no better in children in the same age guideline categories was surprising. It makes intuitive sense that adherence would be higher if parents only need to reinforce and monitor one time limit for all their children, particularly given these are 24‐h behaviours that by definition are time limited (Gába et al., 2020; Olds et al., 2012). In our sample, most of the families with children in the same age category had older children, and we know from research in this cohort and other studies that PA tends to decrease with age (Australian Institute of Health and Welfare, 2020; Olds et al., 2018; Roman‐Viñas et al., 2016; Tooth et al., 2019). This may possibly explain the result. For PA, adherence was better in younger children if they had no older or same‐aged sibling and better for older children with a younger sibling. The finding in the sensitivity analysis that both age and sibling effects were occurring reinforces the importance of including multiple children from each family when investigating guidelines.

Our study is novel in that it included multiple children in a family, unlike most research that only includes one child from a family. Our findings show that adherence is complicated. We speculate that by not examining multiple children in a family, research may be missing the potentially important role that age plays within family‐level adherence and could also play in the associations between movement guidelines and child development outcomes (Tooth et al., 2021). We showed a high percentage of discrepant adherence within families, with 44% and 36% of families with children of different ages having some meet/some fail ST and PA guidelines, respectively. By not accounting for these complexities of adherence, results may not adequately reflect what is happening within families.

There is substantial public health concern about children's poor adherence to 24‐h movement guidelines (Friel et al., 2020; Olds et al., 2018; Rollo et al., 2020; Roman‐Viñas et al., 2016; Sampasa‐Kanyinga et al., 2017; Tapia‐Serrano et al., 2022), particularly considering the clear mental health, physical health, cognitive and health‐related quality of life benefits (Rollo et al., 2020; Sampasa‐Kanyinga et al., 2017). There are calls for better health promotion and prevention campaigns and support to be offered to children and adolescents to assist them to meet guidelines (Friel et al., 2020; Rollo et al., 2020). For very young children however, parents do need to take responsibility for helping them develop healthy screen use habits, as they are not capable of regulating their own screen use (Morawaska et al., 2023). As shown in the current study, in families where children are of different ages, adherence is mixed. Australian parents are generally aware of preventive health behaviours (Baker et al., 2020), yet adherence is poor, not only in children but also in many adults themselves. This may reflect parent confidence in their ability to support and reinforce guideline adherence, particularly for PA (Rhodes et al., 2019). Some parents may have a low level of awareness of health behaviour guidelines (Sawyer et al., 2014), or difficulties understanding guideline messages, for example, due to health literacy (Hollman et al., 2022). Parents may also find guidelines unrealistic, too difficult or challenging to reinforce, despite guilt (Hollman et al., 2022) and good intentions (Rhodes et al., 2019). Parents may find it easier to implement a one‐size‐fits‐all rule for all their children, resulting in some children meeting guidelines while others do not. Our earlier study of ST adherence showed that of the families with children in different age‐based categories where only some met the guidelines, 92% of children aged 2–4 years exceeded the guidelines: They were meeting the guidelines of their 5‐ to 12‐year‐old siblings (Tooth et al., 2022).

Recommendations for helping parents manage guideline adherence include acknowledgement that 24‐h movement behaviours need to be considered together, ensuring that guidelines use simplified language and are tailored to a child's particular circumstances (e.g., age, gender, physical ability and socio‐economic status), quantification of what adherent behaviours are and relatable examples of how to integrate adherent behaviours into daily life (Friel et al., 2020; Hollman et al., 2022; Jarvis et al., 2021; Latimer‐Cheung et al., 2016; Olds et al., 2018; Roman‐Viñas et al., 2016; Tapia‐Serrano et al., 2022). Parenting resources and guideline messaging need to acknowledge the challenge parents face in managing different guideline recommendations in the family. We further recommend that research into guideline adherence includes multiple children in the family to improve generalisability.

### 4.1 Strengths and limitations

Strengths were the inclusion of multiple children within the family, the broadly representative national sample of Australian families and children and wide range of covariates. Limitations included using parent report to record ST, PA and sleep, including social desirability bias. It may be difficult for parents to reliably know how much PA and ST their children are doing, particularly outside the home. While the PA instrument in our study has been found to produce estimates that correlate with accelerometer data in epidemiological studies involving adolescents (Prochaska et al., 2001), the measure may not be comparable with research using more narrow age bands or different time scales for determining adherence (Gomes et al., 2017). The mothers who participated in MatCH were 37–43 years, which may introduce bias as adherence may differ in families with younger mothers. The MatCH study sample had higher socio‐economic status compared with similarly aged Australian women, which limits generalisability. The MatCH data were cross‐sectional, which limits consideration of causality.

## 5 CONCLUSIONS

In conclusion, overall adherence to 24‐h movement guidelines may be more challenging for families that have children spanning age‐based guideline categories. Information, messaging and implementation of strategies to promote and enhance meeting ST, PA and sleep guidelines in children need to include practical advice for families with multiple children.

## AUTHOR CONTRIBUTIONS

**Leigh R. Tooth:** Conceptualization; methodology; funding acquisition; writing—original draft; writing—review and editing; investigation. **Gregore I. Mielke:** Conceptualization; writing—review and editing; data curation; formal analysis. **Katrina M. Moss:** Conceptualization; methodology; data curation; formal analysis; writing—review and editing.

## CONFLICT OF INTEREST STATEMENT

The authors declare that they have no conflicts of interest.

## ETHICS STATEMENT

Both studies were approved by the Human Research Ethics Committees at the University of Queensland and University of Newcastle in Australia. Mothers provided informed written consent for all surveys in both the ALSWH and MatCH studies. All data collected in both surveys were provided by the mothers.

## Supporting information


**Table S1.** Covariates and their measurement

## Data Availability

The datasets supporting the conclusions of this article are available from the Australian Longitudinal Study on Women's Health (ALSWH) (https://alswh.org.au/for-data-users/applying-for-data/). ALSWH survey data are owned by the Australian Government Department of Health and Aged Care. Due to the personal nature of the data collected, release by ALSWH is subject to strict contractual and ethical restrictions. De‐identified data are available to collaborating researchers free of charge where a formal request to make use of the material has been approved by the ALSWH Data Access Committee. The committee is receptive of requests for datasets required to replicate results. Information on applying for ALSWH data is available from https://alswh.org.au/for-data-users/applying-for-data/. Mothers and their Children Health (MatCH) sub‐study data, including weights to enable comparisons with the Australian population, will become available later in 2023 with access through the same process described above.
