# Supplementary material for: Adherence to 24‐h movement behaviour guidelines in families with multiple children
Source: Child Care Health Dev. 2023 Dec 17;50(1):e13213. doi: 10.1111/cch.13213 (PMC10952476; doi:10.1111/cch.13213)
Supplement: Supplementary file 1 — Table S1. Covariates and their measurement [file CCH-50-0-s001.docx]

**Supplementary Table: Covariates and their measurement**

| **Covariate** | **Measurement** | **Source or Reference, if applicable** |
| --- | --- | --- |
| Area of residence | Major city, inner regional, outer regional, rural/remote | Determined from participant address.  Categorised by: Glover J, Tennant S. Remote areas statistical geography in Australia: Notes on the accessibility/remoteness index for Australia (ARIA+ version). 2003. Available at: https://www.semanticscholar.org/paper/Remote-Areas-Statistical-Geography-in-Australia%3A-on-Glover-Tennant/cbfb7493312dee029e43d29e0795e4bd10d9bc16. |
| Partnership status | Partner (married/defacto), no partner (single, widowed, divorced) | From ALSWH survey, https://alswh.org.au/for-data-users/data-documentation/surveys/ |
| Highest educational qualification | Up to grade 12, trade/certificate/diploma, degree/higher degree | From ALSWH survey, https://alswh.org.au/for-data-users/data-documentation/surveys/ |
| Body mass index | Underweight/healthy weight (BMI ≤25), overweight (BMI 26-≤30), obese ((BMI >30) | From ALSWH survey, https://alswh.org.au/for-data-users/data-documentation/surveys/  Categorised by: Obesity: Preventing and managing the global epidemic. World Health Organization. 2000. Available at: https://apps.who.int/iris/handle/10665/42330. |
| Level of physical activity | Based on metabolic equivalents Nil (0-<40 Met.mins/week), low (40-<600 Met.mins/week), moderate (600-<1200 Met.mins/week), or high (≥1200 Met.mins/week) | From ALSWH survey, https://alswh.org.au/for-data-users/data-documentation/surveys/  Categorised by: Brown WJ, Ford JH, Burton NW, Marshall AL, Dobson AJ. Prospective study of physical activity and depressive symptoms in middle-aged women. *Am J Prev Med* 2005;29(4):265-272. doi: 10.1016/j.amepre.2005.06.009. |
| Depressive symptoms | 10-item Centre for Epidemiology Studies Depression Scale-10. Score of ≥10 indicates depressive symptoms | From ALSWH survey, https://alswh.org.au/for-data-users/data-documentation/surveys/  Categorised by: Andresen E, Malmgren J, Carter W, Patrick D. Screening for depression in well older adults: evaluation of a short form of the CES-D. *Am J Prev Med* 1994;10:77–84. |
| Sex of child | Female/Male | From MatCH survey, https://alswh.org.au/match/match-data/ |
| Electronic equipment in the child’s bedroom | Yes/No | From MatCH survey, https://alswh.org.au/match/match-data/ |
